# On the Beat Detection Performance in Long-Term ECG Monitoring Scenarios

**DOI:** 10.3390/s18051387

**Published:** 2018-05-01

**Authors:** Francisco-Manuel Melgarejo-Meseguer, Estrella Everss-Villalba, Francisco-Javier Gimeno-Blanes, Manuel Blanco-Velasco, Zaida Molins-Bordallo, José-Antonio Flores-Yepes, José-Luis Rojo-Álvarez, Arcadi García-Alberola

**Affiliations:** 1 Cardiology Service, Arrhythmia Unit, Hospital General Universitario Virgen de la Arrixaca, El Palmar, 30120 Murcia, Spain; estrella.everss@urjc.es (E.E.-V.); zaidamb23@gmail.com (Z.M.-B.); arcadi@secardiologia.es (A.G.-A.); 2 Department of Signal Theory and Communications, Miguel Hernández University, Elche, 03202 Alicante, Spain; ja.flores@umh.es; 3 Department of Signal Theory and Communications, University of Alcalá, Alcalá de Henares, 28805 Madrid, Spain; manuel.blanco@uah.es; 4 Center for Computational Simulation, Universidad Politécnica de Madrid, Boadilla, 28223 Madrid, Spain; 5 Department of Signal Theory and Communications, Rey Juan Carlos University, Fuenlabrada, 28943 Madrid, Spain

**Keywords:** QRS detection, ECG, long-term monitoring, Holter, 7-day

## Abstract

Despite the wide literature on R-wave detection algorithms for ECG Holter recordings, the long-term monitoring applications are bringing new requirements, and it is not clear that the existing methods can be straightforwardly used in those scenarios. Our aim in this work was twofold: First, we scrutinized the scope and limitations of existing methods for Holter monitoring when moving to long-term monitoring; Second, we proposed and benchmarked a beat detection method with adequate accuracy and usefulness in long-term scenarios. A longitudinal study was made with the most widely used waveform analysis algorithms, which allowed us to tune the free parameters of the required blocks, and a transversal study analyzed how these parameters change when moving to different databases. With all the above, the extension to long-term monitoring in a database of 7-day Holter monitoring was proposed and analyzed, by using an optimized simultaneous-multilead processing. We considered both own and public databases. In this new scenario, the noise-avoid mechanisms are more important due to the amount of noise that exists in these recordings, moreover, the computational efficiency is a key parameter in order to export the algorithm to the clinical practice. The method based on a Polling function outperformed the others in terms of accuracy and computational efficiency, yielding 99.48% sensitivity, 99.54% specificity, 99.69% positive predictive value, 99.46% accuracy, and 0.85% error for MIT-BIH arrhythmia database. We conclude that the method can be used in long-term Holter monitoring systems.

## 1. Introduction

The main purpose of cardiac monitoring is the detection of cardiac diseases with transient manifestations or symptoms. The most frequently used device to continuously record the heart’s rhythm during daily activities is the 24-h Holter, in which patients are continuously monitored during one day through a series of electrodes attached to the skin and while performing their daily activities. This procedure can be also carried out during longer periods, such as seven days, 15 days, or 21 days, thus recording what we refer to as Long-term Monitoring (LTM). In previous research, LTM has been proven to be an important diagnostic tool, since it can detect several diagnostic indicators that are not always present in shorter monitoring periods (specially for transient and very infrequent electrocardiographic abnormalities). For this reason, recent research is increasingly focusing on LTM protocols and systems [1,2,3,4]. Inside this new scenario, one of the most important challenges is the extraction of different parameters that can be used to improve the diagnostic accuracy. Using traditional protocols and signal processing techniques, the massive amount of data that the clinicians must analyze and validate for each patient represents a time-consuming challenge. To solve this problem, new protocols and new more advanced signal processing techniques are required. Beat detection is one of the most relevant electrocardiogram (ECG) processing techniques because finding the exact location of QRS complexes (i.e., the signal deflections corresponding to ventricular depolarization) enables the segmentation of the ECG into its sub–waves, which is often a basic step for multiple and more complex algorithms [5]. For this reason, R-detection is usually done in the initial stage before beat-feature extraction and beat classification [6]. An example of the importance of the correct heartbeat detection is its application to the study of changes in the heart rate, which is a key parameter to analyze many heart diseases, such as arrhythmias, and arrhythmic risk assessment of patients, among others [7].

Although the detection of the QRS complex has been extensively studied over the years, it is still a field under development. The reason is that the detection of the R-wave in the ECG is an apparently very simple problem, which can be tackled from many different viewpoints. Initially, the development of accurate methods was the point of interest [8,9], but, currently, research mainly focuses on the design of novel methods usually based on modern processing techniques. Despite the application of advanced data processing methods, the performance of the new approaches seldom overcome the former ones. In fact, in many cases, some recent techniques are applied in combination with traditional methods such as the Pan and Tompkins algorithm (PT) or frequency transforms [9,10,11,12]. Accordingly, huge amounts of contributions have been reported, the literature in this area is large and complicated, and the task of determining which is the best suited or the more efficient algorithm among the available ones has become difficult.

Beyond the accuracy of the methods, not all of them can be straightforwardly applied to LTM recordings. The initial constraint is given by the limited computational capacity of current equipment to process the big amount of data resulting from 7- to 21-day ECG recordings. Additionally, the high performance exhibited by current methods is difficult to reach in LTM applications. One of the reasons is the testing methodology, and, despite the algorithms being checked by means of comparison tests against state-of-the-art methods and over public databases, the set size often is limited, which makes some analysis be scarcely representative. Another, and perhaps more important reason, is that the algorithms are sensitive to the dataset, so that the parameter tuning needs to be readjusted depending on the working conditions. Therefore, an R-wave detector for LTM recordings must be only be accurate, but also reliable, robust, and efficient from a computational cost viewpoint, in order to be able to deal with very long sets in the presence of noise and artifacts [13].

Therefore, and taking into account the current situation of the R-peak detection field, the purpose of this work was twofold. On the one hand, we aimed to determine the scope and limitations of the R-wave detection algorithms currently available when they are applied to LTM records. On the other hand, we proposed an accurate and reliable method to be specifically designed for LTM. In order to achieve these targets, we drove both a qualitative and quantitative study by means of a longitudinal and a transversal analysis. The longitudinal analysis aimed to identify and analyze the most commonly used processing blocks in this field, and the results derived from this part of the study enabled the design of an efficient detection system with the most suitable processing blocks. The transversal analysis was carried out to overcome the effect of using different databases and given the dependency of the detector with the dataset used in its original design. This part of the study provided the clues to correctly refine the parameters of the different elements of the detection algorithm. The transversal analysis was tackled through comparisons carried out over several public and own large databases.

As a result, we present here a reliable, robust, and efficient R-wave detector for LTM, outpacing the existing ones, and benchmarking it simultaneously over a wide range of databases. To do so, we deeply stress-tested the performance of the most popular published methods, not only over the traditional public (and well known) databases, but also over two trusted and solid new databases of 24-h and 7-day LTM clinical recordings, from Hospital Virgen de la Arrixaca de Murcia. These private databases contain a variety of patient illnesses, as well as different signal noises, lengths, or recording characteristics, such as sampling frequency and number of bits per sample. The developed optimal detector consisted of a new–and–modified PT algorithm based on simultaneous-multilead processing, which outperformed all the tested methods in terms of efficiency and accuracy.

This paper is structured as follows. Section 2 describes the appliances and tools used for signal registering and processing, and then it elaborates on details for all the algorithms and databases used for signal processing. Section 3 includes the different selected methods, while, in Section 4, experiments are applied over our database and the corresponding results are reported. Finally, Section 5 contains the conclusions of all the work carried out.

## 2. Databases

For testing the algorithms and methods analyzed and designed in this paper, several private and public databases have been used. More precisely, a private 24-h Holter database that was manually labeled by experts was used to analyze the optimization of classical algorithms in a high–quality annotated and long-duration set of recordings. After that, the most popular public labeled databases in the scientific literature were used to compare our results with those of others, aiming to account here for possible deviations in the algorithm accuracy due to overfitting to the database used for algorithm tuning. Finally, we worked with another set of labeled 7-day Holter recordings in order to adapt the algorithms to a larger time-scale database. To our best knowledge, there is not a public LTM database manually and beat-by-beat labeled with respect to arrhythmic events, hence we decided to use again the available recordings from precedent studies, which had been carefully revised because their research use. Table 1 shows a summary of the names of the databases and their characteristics.

(1) *vPREDICT Private Database.* The vPREDICT project [14,15] was designed to establish data relationships between electric cardiac signals and sudden cardiac death events using signal processing based indices. Given that these recording were assembled for research purposes, they had been carefully annotated, which made them suitable for quality validation of ECG signal-processing algorithms. This database contains 24-h Holter recordings from 122 heart failure patients in 5 different hospitals. For testing the algorithms and methods in this work, only 16 patients were selected among them, including diverse clinical conditions, namely, two subjects with normal heart function, five with an implanted defibrillator, five with atrial fibrillation, and the last five with frequent premature ventricular contractions. Two different Holter recorders were used, namely, SpiderView former ELA Medical, and now part of Livanova , and SpiderView Plus (former Sorin Group, and now also part of Livanova). The acquisition was made with 3 or 5 sensors, yielding the usual two or three leads in Holter, and the sampling rate was 200 Hz. The electrodes were placed according to SpiderView manual, i.e., in the case of 3-electrode configuration, the electrodes were placed on the sternal manubrium, the xyphoid process and the area of the apex generating a pseudo-II and V5 leads.

(2) *Public Databases.* For decades, Physionet has offered online large physiological collections of signals that have powered the clinical and technical research in many fields. In this work, and in order to evaluate the generalization of the optimization of classical algorithms previously used in the vPREDICT database, six open–access labeled ECG signal databases from Physionet were used:

- MIT–BIH. The MIT–BIH Arrhythmia database contains a representative collection of independently annotated ECG signals, consisting of 48 half-hour excerpts of two–lead 24-h ambulatory ECGs with clinically significant arrhythmias sampled at 360 Hz [16,17]. We chose this classical database due to its widespread and current use [18].
- NSTDB. The MIT–BIH Noise Stress database includes 12 half–hour ECG recordings and three half-hour recordings of three types of noise that are more commonly present in ambulatory monitorization, namely, baseline wander, muscle artifact, and motion artifact. Noise data were made using physically active volunteers with standard ECG recorders, leads, and electrodes, and then adding it to the recordings from the MIT–BIH [19,20]. We selected these data for the wide use of its noise components (e.g., see [21]).
- LTSTDB. The Long-term ST database includes 86 Holter recordings of two or three leads with a duration between 21 and 24 h. This database shows a variety of events of ST segment changes [20,22].
- LTAFDB. The Long Term AF database includes 84 two-lead Holter recordings sampled at 128 Hz with a duration between 24 and 25 h [20,23].
- LTMITBIHDB. The Long-term MIT–BIH database includes 7 Holter recordings between 14 and 22 h [20].
- INCART. St. Petersburg Institute of Cardiological Technics 12–lead Arrhythmia database consists of 75 annotated recordings extracted from 32–half–hour Holter recordings of coronary artery disease patients sampled at 257 Hz [20]. In order to compare our Holter recordings with other Holter segments, we chose this data following other current researchers (e.g., see [24]).

(3) *PREMARIS Private Database.* This database was manually labeled by experts, and it has been used in previous research by our group [25,26]. It includes 84 real cases of 7-day- Holters, in patients suffering from heart failure (HF) and/or left ventricular dysfunction. In order to evaluate the performance of the previously developed algorithms over real LTM recordings, five patients with sinus rhythm were selected from the database. Four out of these five were males. It should be mentioned here that 7-day Holter recordings are registered during the normal daily life of patients, and so these records included not only the usual amount of artifacts and noise in standard ECG, but also strong presence of sensors decoupling and signal losses due to the long exposition. The sampling frequency was 128 Hz.

## 3. R-Wave Detector System

Several types of QRS detector methods have been proposed to date in the literature and in the commercial systems. The most usual family of algorithms used for 24-h Holter monitoring is based on waveform analysis principles. These algorithms use signal processing techniques to reduce noise as well as to emphasize relevant signal elements through a number of computational strategies. In this work, we scrutinize the performance of the most commonly used R-wave detector systems applied to 24-h Holter recordings, and specially how they behave when they are moved towards LTM scenarios. Towards that end, we present in this section a compiled architecture of the best knowledge in the literature. Waveform algorithms applied in literature are generally categorized into two subsets. The first one incorporates all those methods using multiple leads that are recorded through parallel acquisition. Examples of these multilead processing techniques are those using Principal Component Analysis (PCA), Independent Component Analysis (ICA), or Linear Discriminant Analysis (LDA) [27]. The second subset of algorithms treats each lead individually, and they apply filters in order to reduce the noise and enhance the relevant signal components. Examples of this second type include the very well-known PT, as well as the Hamilton–Tompkins (HT) and the Chouhan methods [9,28,29,30]. Table 2 shows the accuracy rates benchmarked over the MIT–BIH of relevant methods on the literature, such as Continuous Wavelet Transform (CWT) [31], Short Time Fourier Transform (STFT) [32], Hilbert Transform (HiT) [33], or Hilbert–Huang Transform (HHT). These reported values should be considered as the standard target accuracy also for LTM scenarios, but the methods should also be competitive in terms of computational cost, taking into account the dramatically longer signal duration.

**Figure 1:**
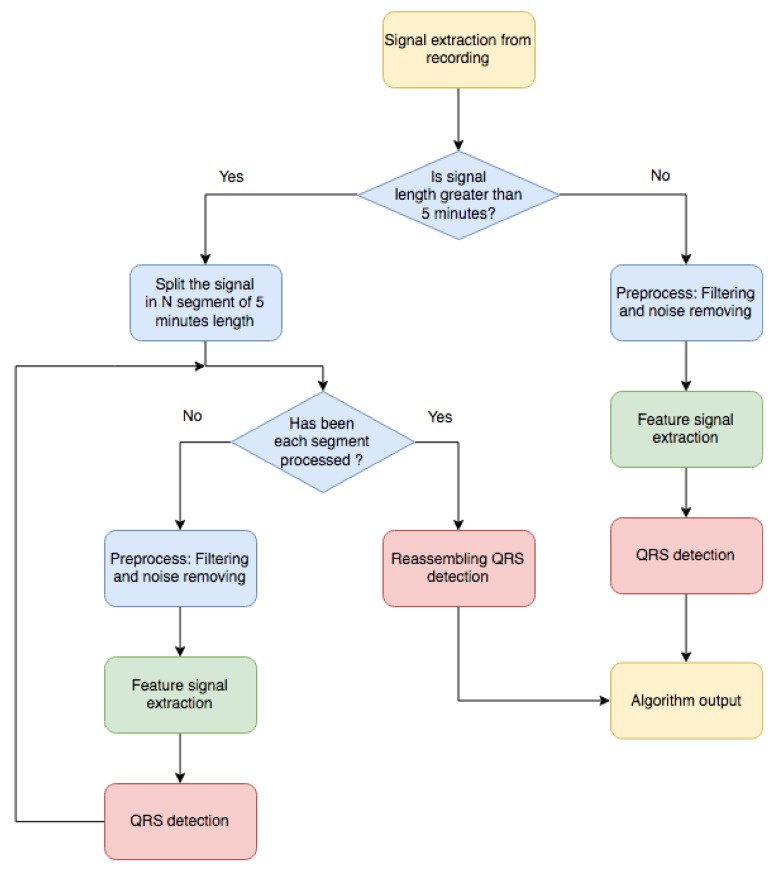
Main stages of the QRS detection algorithms proposed in this work. The yellow boxes are inputs/outputs, the blue boxes represent the preprocessing stage, the green boxes represent the feature signal extraction stage, and the red boxes represent the QRS detection stage.

Our literature review highlighted the common stages for R-wave detection in commercial algorithms [9,10,15,28,29,36]. Roughly speaking, three steps are generally applied (see Figure 1). First, *Signal Preprocessing* is usually performed, in which the signal is adjusted to fit a certain common standard by removing noise, baseline wander, and undesirable artifacts. To do so, different types of filters are applied, such as low–pass, high pass, and notch filters. In a second stage, usually known as *Feature Signal Extraction*, a much stronger and sometimes uncompromisingly processing is performed in order to isolate the QRS complexes for their better detection. At this stage, the focal point is to detect the presence of the QRS complexes by the creation of a new signal, a so-called feature signal, where the QRS complexes are enhanced in order to ease their detection in the next stage. In the final *R-wave Detection* stage, the R-waves are situated by using an adaptive–threshold based on statistical and temporal properties of the feature signal. Many algorithms are proposed in the literature for this final and critical steps, although the main differences in terms of accuracy and computational cost are found in the Feature Extraction stage. Here follows a deeper detailed description of each of the stages including a short description of the methods present in literature, as well as new adapted methods developed in this work to enhance the performance. The reader should notice that no exhaustive description or full enumeration of all the reported methods are included in this work, as over 500 papers are released yearly in this topic, but rather the most relevant and effective methods are described, implemented, benchmarked, and summarized hereafter.

### 3.1. Signal Preprocessing Stage

Preprocessing is the first stage of the algorithm, and decisions taken at this point will impact all the subsequent blocks. In our specific application for LTM, a prior segmentation process is applied for computational convenience. Segmentation is the process of dividing the ECG record into smaller segments to be processed efficiently [18,37,38], according to standard computational resources. Although segmentation is not always included or mentioned in the literature, as it has no impact on standard clinical ECG records of few seconds, it is of relevant impact in LTM implementations. As a token, the reader should be aware that a 3-lead signal, sampled at 200 Hz with 15–bit resolution and during 48 consecutive hours, creates a single file of about 100 MB. This file size becomes intractable when it has to be handled with intensive processing techniques, and commercial devices do not always have enough resources.

**Figure 2:**
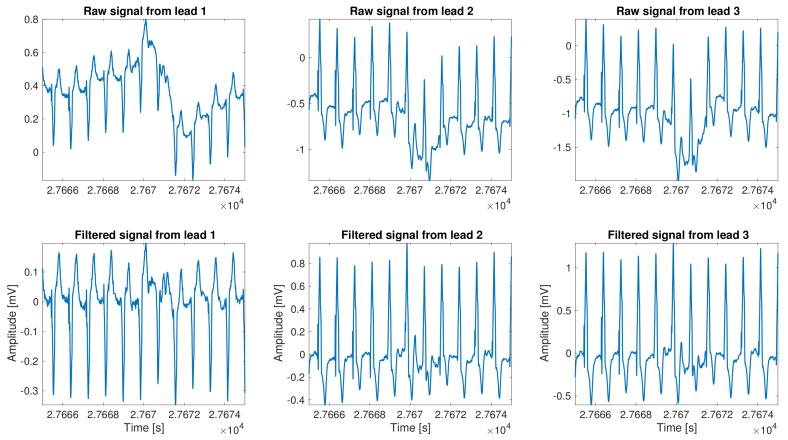
Example of the signal preprocessing stage over a segment of 3-lead signal from vPREDICT database.

As a part of the standard preprocessing, denoising, baseline wander, and signal detrending are very well described in literature, and frequently built-in in almost any commercial or research system. The removal of these undesirable components is generally referred to as preprocessing, although as we mentioned before it could also include some other signal accommodation. The most common types of noise present in the ECG methodology are power line interference, other device electromagnetic interference, muscle artifacts, breathing induction, and artifacts related to decoupling of sensor interfaces. The relevance of all these possible elements have a capital impact on LTM due to the unfeasibility to keep the recording device in a noise–free environment and connected to ground, as the device is portable and the patient should be running his normal life during the recording period. Our implementation incorporated the following algorithmic stages: cubic spline detrend baseline, power line and harmonic notch filtering, a low–pass filter to remove muscle artifact and other high frequency interferences, and, finally, a filter based on the time–local variance, which is used to remove the patient–electrode motion artifacts [13]. An example of the result of this stage is shown in Figure 2.

### 3.2. Feature Signal Extraction Stage

The next step after preprocessing is Feature Signal Extraction. At this stage, the focus is not to retain the ECG morphology for diagnostic purposes, but rather to undoubtedly identify the region where the QRS complexes are. A number of algorithms have been proposed that compute the feature signal in many ways. We can classify them all in two families attending to the way it is being processed. The first family could be called Matrix Methods. These methods leverage on the multiple available leads to enhance the QRS by applying very well-known multichannel processing techniques, such as PCA and ICA [39,40,41,42,43,44]. The second family could be generally named as Digital Filtering methods, and it includes PT, Root Mean Square (RMS), and combinational methods. All the methods in this family share the use of filtering techniques to enhance the QRS against the rest of the elements present in the ECG signal.

PCA is one of the most relevant Matrix Methods since it was first introduced [45], and it consists of a multivariate linear conversion that transforms the original signals into a new coordinate system where signals are orthogonal. This transformation may eventually highlight relevant elements, as the QRS, in one of the output signals if the input signals incorporated different projections of these elements. As a result, this processing could reduce both noise and data dimensionality, selecting only the output signals with relevant information. PCA is often used in a number of denoising processes, and it has also shown to be appropriate for multilead Holter recording analysis, where different noise sources distort the measured signal. To perform this processing, the covariance matrix of available leads is calculated, as well as its eigenvectors. As a second step, a transformation is applied ensuring orthogonality, and the output new signals are sorted according to their eigenvalues, which ensures to retain the largest variance components. The eigenvector associated with the largest eigenvalue corresponds to the largest explained variance from the decomposition [46], which usually makes the projection of all the leads onto that direction the most suitable to be used as a feature signal for this stage. Once it has been obtained, it is often squared to highlight the R-peak magnitude, which usually enables an enhancement on its presence. The second technique applied under the Matrix Methods is the ICA, which also allows us to represent a set of signals as a combination of statistically independent components. Its main purpose is the extraction of signals from a signal mixture model created under unknown conditions. This situation mirrors and matches the real fact of the different projections of the QRS in multiple lead signals. In order to apply this technique, the signals must meet certain conditions, namely, the original signals are assumed to be independent among them, and they follow a non-Gaussian distribution. Different from PCA, it is not clear which component is more relevant than another in ICA [47], and in this work we used the heuristically accepted approach of sorting the relevance of the projection directions with the descending kurtosis of the projected output.

Entering now in the Digital Filtering methods, the PT algorithm [9] designed to provide QRS complex detection in real time is widely used, and it may be the most benchmarked method of all the ones reported in the literature. This method works on a single lead, and in the case of having more than one lead, the one with lowest noise content is selected. The signal is then filtered, first with a narrow-band-pass filter, and then with a derivative, which emphasizes the QRS segment. The resulting signal is squared to boost the amplitude of R-peaks, and a mean filter is applied with a sliding window in order to integrate the signal. Finally, the R-peak detection is done with the application of a threshold over the amplitude of the before computed signal. Two main drawbacks of this PT algorithm method should be mentioned. First, the initial selection of the less–noisy lead is crucial, having a relevant impact on final results, and, second, the threshold selection is not always direct or evident, and it has noticeable effects on the final results.

To improve the observed disadvantages involving the PT detector, especially the first one, we proposed and developed a set of new algorithms based on this very well known method. We describe and present here only that provided better results. One of them is the RMS method, which creates a ready–for–thresholding feature signal from a multi–lead Holter recording by just combining all the leads as follows:

(1) $$y_{c}\left[n\right]=\sqrt{\sum_{l=1}^{L}x_{f}^{2}\left[n,l_{i}\right],}$$

where $y_{c}\left[n\right]$ is the feature signal, $x_{f}\left[n,l_{i}\right]$ is the *i*th lead of the pre–processed signal filtered with the PT derivative–filter, and *L* is the number of leads.

Another implemented variation consisted on applying PT derivative–filter to each lead and the result is mixed using the following combinational lead approach:

(2) $$y_{c}\left[n,l_{i}\right]=x_{f}\left[n,l_{i}\right]\sqrt{\prod_{j=1,i\neq j}^{L}x_{f}\left[n,l_{j}\right].}$$

This last implementation of a combinational method consists of the evaluation of the feature signal for R-peak detection on each lead and to jointly assess the detection based on a certain algorithm applied to all of them simultaneously. Four different possibilities were calculated and benchmarked. The first one is called the And method, in which all the detection vectors are combined by using the and logical function. The second one is called the Polling (Poll) combination, and, in this method, if a peak is detected in the majority of the leads, then it is tagged as a valid peak. The third one is called Simple Coupling (SC), and it is based on the And method, but to report an R-peak as valid, it must be present in at least two leads. The last one is known as Or, and all the detected R-peaks are combined by the use of the or logical function. As the physical location of the R-peak may differ slightly among leads, a tolerance window is implemented around the peaks as far these combinational methods are concerned. Maximum tolerance was set to 10 ms based on the clinical knowledge and empirical review. Additionally, a refractory period for R-peak detection was set in order to avoid artifact detection and other prominent false positive detections, such as prominent T–peaks.

**Figure 3:**
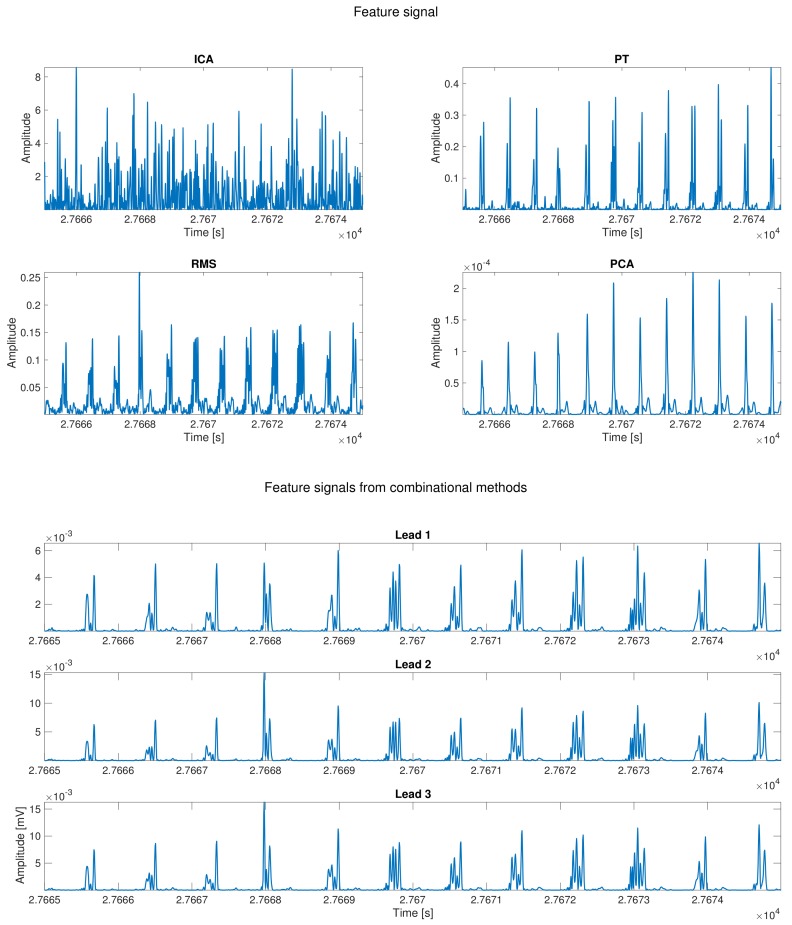
Computed feature signals for each proposed method. The upper four panels show the feature signal for the next methods: ICA, PCA, PT, and RMS. In the lower panel, the feature signals from the combinational methods are presented.

Figure 3 shows the different feature signals computed for each method presented in this work using the signal shown in Figure 2.

### 3.3. R-Wave Detection Stage

This subsection describes the method followed to finally locate the R-peak in the ECG previously determined region. We implemented a thresholding method as usual in the literature [10,15,29,36]. The threshold is defined as α·y+β·std(y), where *y* is the feature signal that was calculated according to the methods seen before, and it is applied to a widen by 75 ms of the preselected QRS segment.

**Figure 4:**
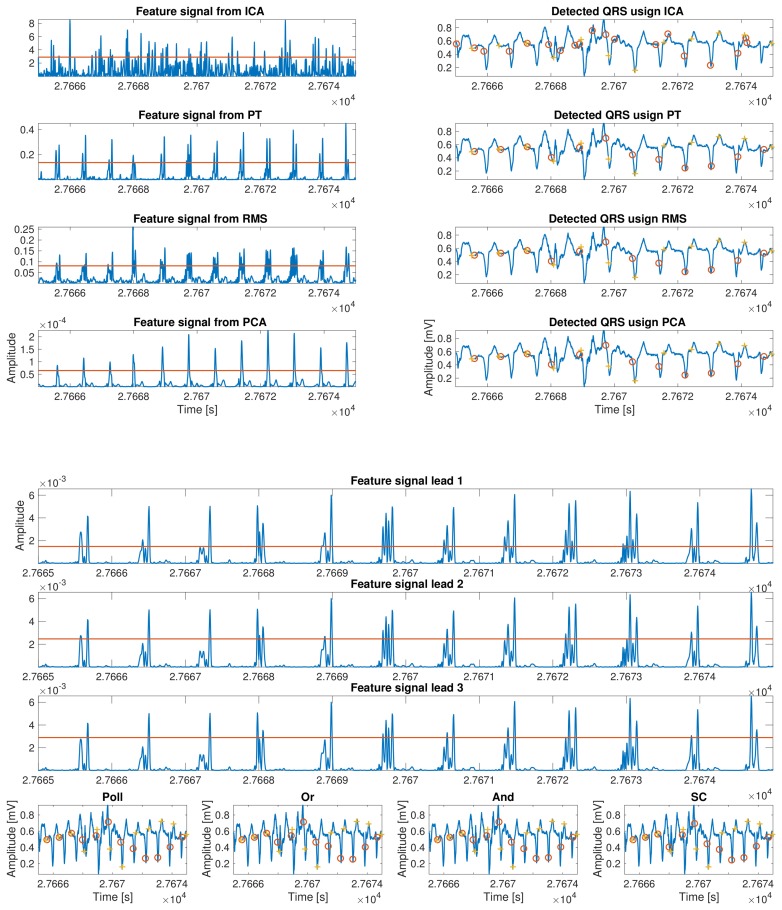
Thresholding process and peak detection for each proposed method. Panel one shows the feature signal, the threshold, and the peaks detected for the next methods, ICA, PCA, PT, and RMS. In panel two, the feature signal, the threshold, and the peaks detected for the combinational methods are presented. The yellow crosses shown the real peak presented in the signal, and the red circles shown the peak detected by each method.

Once all the segments that contain an R-wave are determined, the detection is carried out using an algorithm with two steps. First, we implemented a coarse detection stage where the algorithm searches the maximums of each of the previous regions candidates. This allows for a specific detection of the R-waves using the following equation: $Prepeak\left(r\right)=max(|\left(x_{f}\left[r,l\right]\right)|)$, where *r* refers to the previously calculated segments and $x_{f}\left[r,l_{i}\right]$ stands for the *i*th lead of featured signal computed before. Second, we implemented a fine detection stage, where the algorithm looks for the maximum value in a window centered in each pre–detected peak over a softer filtered version of the initial input signal. In Figure 4, an example of peak detection for each proposed method is shown.

## 4. Experiments and Results

The algorithms presented in the previous section need to be tuned by setting different parameters in order to maximize their performance. This section is divided into three subsections. The first subsection correspond to the so-called *Longitudinal Optimization*, and includes the free-parameter optimization on the private database vPREDICT. The results of this step allows the best algorithm preset to be used here and after. In the second subsection, so-called *Transversal Optimization*, the knowledge acquired in the previous section is applied to different public databases from the Physionet repository. This application provides a three-fold outcome. First, it allows us to check the performance versus different databases and to make the model independent from the used database. Additionally, it allows us to benchmark versus well known databases, and improve the performance over a second parameter adjustment. The last subsection, described as *LTM Application*, extends the resulting algorithm over our LTM database and evaluates the different inaccuracies arising in the significant different environment of 24-h to 7-day Holter monitoring.

The throughput analysis of the system relies on the statistics of detections achieved by the algorithm, which can be either correct or incorrect. The study of detections only provide information about the ability of detecting R waves, but it tells nothing about the failures to detect QRS complexes. For this reason, the statistic of the non-detected events must also be incorporated. Regarding detections, a correct match of the algorithm is a detection that falls within the range of a short interval around an annotated QRS, which is referred to as a True Positive (TP). Conversely, a wrong detection is a detected QRS complex that is not corresponding to an actual one; in this case, the detected event is labeled to be a False Positive (FP) detection. Regarding the non-detected events, the failure to detect a QRS stands for a wrong non-detection that is labeled as a False Negative (FN). However, the non-detection of any event between consecutive R waves, i.e., no FPs in between two QRS complexes, stands for a correct match of the algorithm, and it is then classified to as a True Negative (TN). The benchmark comparison is then performed using the classical figures of merit, namely, *Sensitivity (Sen)*, *Specificity (Spe)*, *Positive Predictive Value (PPV)*, *Accuracy (Acc)*, and *Error*, described as follows:

(3) $$Sen=\frac{TP}{TP+FN},$$

(4) $$PPV=\frac{TP}{TP+FP},$$

(5) $$Spe=\frac{TN}{TN+FP},$$

(6) $$Acc=\frac{TP+TN}{B},$$

(7) $$Error=\frac{FP+FN}{B}.$$

In summary, we should mention at this point that standard definition of SPE requires the existence of True Negatives, and, for benchmarking purposes, we have slightly re-defined this very well-known statistic as mentioned for the R-wave detection setting. Thus, the results of SPE should be considered with caution as this new parameter does not reflect exactly the standard definition in other environments.

### 4.1. Longitudinal Optimization

LTM presents two main differences compared with standard ECG records. One of them is its higher relevance as a consequence of the uncontrolled environment in contrast to ambulatory ECG recordings. Another difference is the time length of LTM recordings compared with usual ambulatory recordings. This can have a twofold impact because it is possible to implement techniques that take advantage of the record length in order to improve the processing, for instance by statistical denoising through averaging, but also digital processing may collapse the existing commercial devices. In order to approach this second aspect, the first part of this subsection evaluates the required *Segmentation Optimization* for processing feasibility. Additionally, a *Threshold Optimization* process is included to set the minimum size of the most accurate level of peak detection that should be considered in the subsequent processing steps.

**Figure 5:**
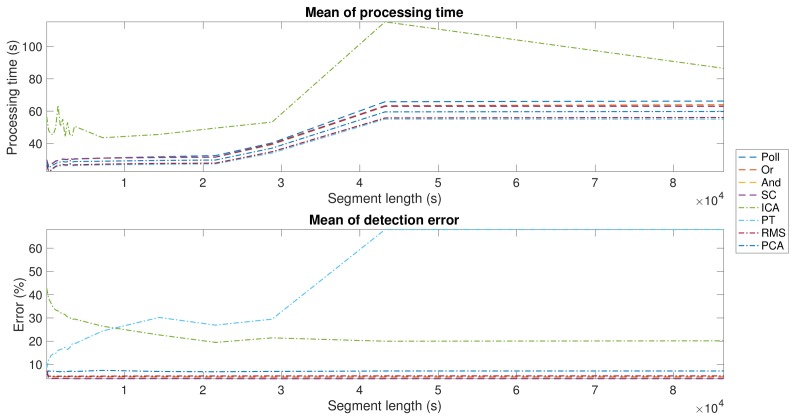
Mean detection errors and processing times for different segment lengths in the proposed algorithms.

**Figure 6:**
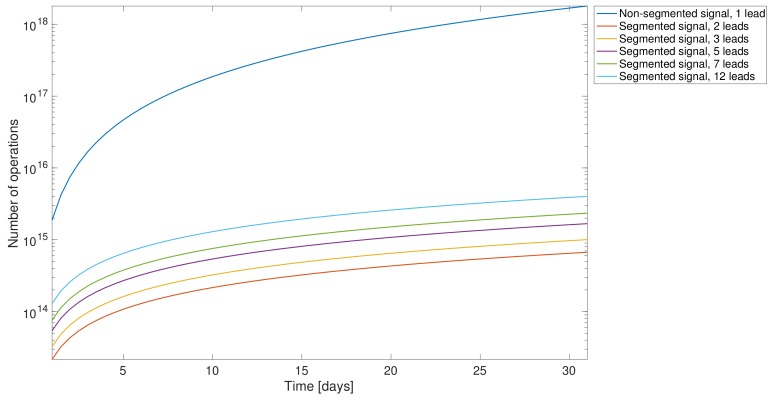
Estimated computational complexity in terms of the number of operations per recording and of its duration (from 1 to 31 days), and for different number of leads. The dark blue line represents the number of operations of a complete single-lead signal, and the others show different lead configurations when including the segmentation stage.

**Segmentation Optimization**. As described before, segmentation is a relevant part of any R-wave detection algorithm, as a suboptimal election of the segment length can cause a heavy computational load and make the algorithm unsuitable for LTM scenarios. For that reason, the aim of this first experiment was to determine the adequate segment length that achieves the minimum processing time. For this objective, a subset of the vPREDICT database was selected and an intensive analysis from 1-min to 24-h segments on 5-min steps was preformed. As shown in the upper panel of Figure 5, all the algorithms exhibited similar behavior in terms of processing time, except for ICA being instable in short segments and divergent above 8-h segments. The errors shown in the lower panel showed a stable performance over the segment lengths, except for PT and ICA, where errors were much higher than in the other methods. In ICA, the performance enhancement was directly related to the segment length, while PT showed the opposite behavior. The best segment lengths and the results for each method are shown in Table 3. Our segmentation approach allowed us to reduce the computational complexity of the problem. Our algorithm is based on the conventional PT algorithms, and it uses the squared-sample operation to build the feature signal. For this reason, the PT complexity has an order of magnitude about $n^{2}$, where *n* is the number of samples of the whole signal. The use of segmentation reduces the complexity to about $S\cdot n_{seg}^{2}$, where *S* is the number of segments, and $n_{seg}$ is the number of samples in a single segment. Figure 6 shows the computational complexity in terms of the number of operations carried out. We compared the following scenarios: (i) our modified PT algorithm applied to one single lead without any segmentation processing, meaning that the application was applied to the whole signal; (ii) in all of the other analysis, our modified PT was applied to various leads simultaneously and including also segmentation. It was proven earlier that segmentation enhanced the computational performance.

**Threshold Optimization**. The aim of the next experiment was to tune the R-wave detection threshold. In order to obtain the best threshold for each proposed algorithm, we implemented a *K*–fold cross–validation with K=4. The results of this experiment are shown in Table 4 for each proposed method, showing that the algorithms with lower detection errors are *Polling* and *And*. We selected the Polling method because it was the best algorithm in terms of error and processing time.

**Optimal R-peak Detection Method**. According to previous experiments, the Polling method outperformed all the other implemented ones. It standouts in terms of computational time, as well as in detection accuracy. The selected algorithm, over vPREDICT and MIT–BIH, presented quite similar results in both cases and in line with algorithms in the literature, as it can be observed in Table 5.

### 4.2. Transversal Optimization

In this subsection, we checked the method independence from the database used during the tuning process. To do so, we first tested on the MIT–BIH, and after that on the rest of available databases.

For better performance and model optimization, some empirically found improvements were applied, which are summarized next: (1) the low-pass-filter cut-off frequency was set to 25 Hz; (2) the refractory period was adjusted according to empirical findings; and (3) the amplitude threshold was set to be updated every three seconds for a closer signal follow–up. These updates required a new *K*-fold cross-validation process, which was applied to MIT–BIH, and it was established K=12. The results were now α = 3 and β = 0.5, and the averaged error was 0.85%.

**Figure 7:**
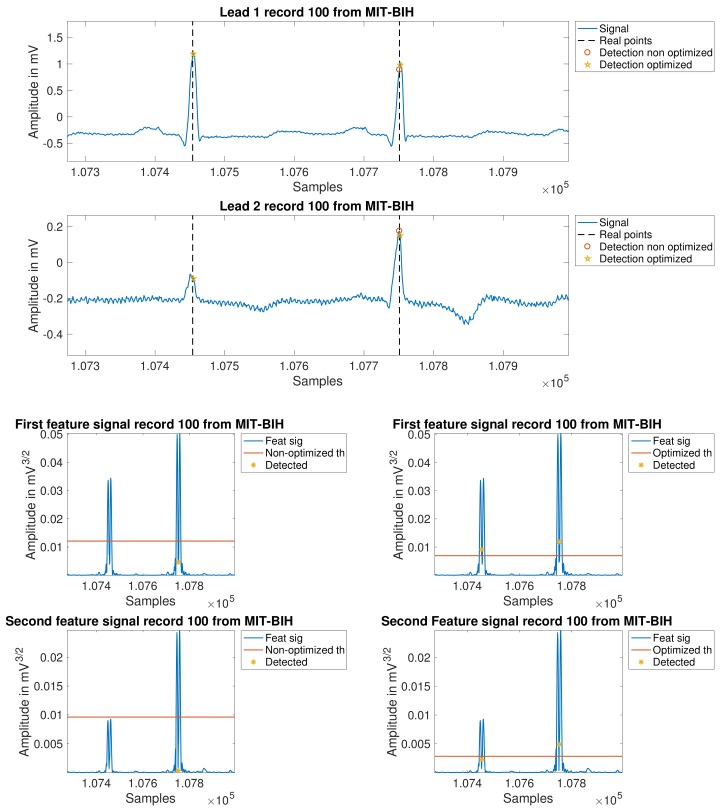
Comparison between the non-optimized and the optimized algorithm in record 100. The two upper panels depict leads 1 and 2. The four lower panels depict the feature signals for both leads.

In Figure 7, the improvements can be scrutinized on several examples. The first two plots depict leads one and two for record 100. In these panels, the reader can see clearly how the new processing is able to closer follow the signal evolution, so that the detection is possible even in the second lead, where it is found a relevant difference on the level of the signal form one beat to another. The following four panels also show the same concept, but now overprinted at two intermediate feature signals, without applying the last mentioned improvements (left) and after applying it (right). Table 6 details these improved results on a per-patient basis.

For testing purposes, the algorithm was also applied to the other databases described earlier, namely, NSTDB, LTSTDB, LTAFDB, LTMITDB, INCART, and vPREDICT. As an example of the resulting performance, Table 7 shows the outcome for the NSTDB. The high performance values obtained show that the algorithm is robust against strong noise. Table 8 additionally summarizes the behavior on all the non-LTM databases used in this paper. It is remarkable that the algorithm settings cope well with all the databases simultaneously, making it a valid algorithm to use in clinical environments, since it has proven its efficacy in this wide variety of cases.

### 4.3. Application to LTM Recordings

After the algorithm has been designed, tuned, and proven accurate over a number of short-term databases, we finally evaluated its behavior in LTM recordings. To do so, the PREMARIS database was used. PREMARIS contains a set of 84 archives of 7-day recordings from patients suffering of HF with left ventricular dysfunction. The details of this database have been introduced previously in the literature [25]. The computational cost of computing over half a million beats on each record suggested to evaluate a subsample of five records of this database. Table 9 summarizes the results over these LTM records. The first finding is a higher number of errors compared with the 24-h recording signals in absolute terms. The reason for this could be found in the difficulties for the user to keep the same care level about the system over the seven days, hence inducing higher noise and probably worst electrodes contact.

**Figure 8:**
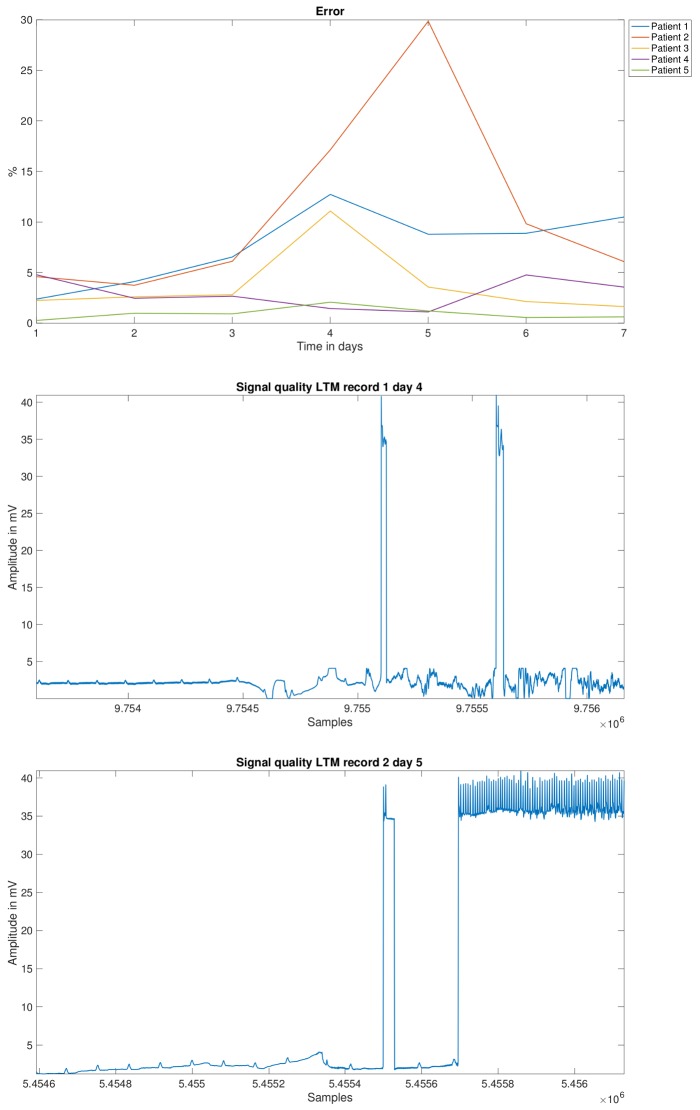
Evolution of the detection error for each LTM patient across the test duration (**up**), and examples of noise types present in 7-day Holter recordings (**middle** and **down**).

In order to explore this speculation, the detection error evolution was plotted as a function of time for all the cases, which allowed a visual representation of the possible errors produced throughout the days of the test. As seen in Figure 8, upper panel, the evolution of the detection errors shows a clear increase in many cases about the middle of the test (days 3 to 5), and a reduction in less proportion when approaching to its end (days 6 to 7). Partial or complete disconnection of the electrodes were the main causes of error, which is related either to incorrect positioning after disconnection, or to daily physical activity of the patient. Any of these situations debuts in an increase of noise levels in the signal. An example of this is shown in the same figure, in the middle and lower panels.

**Figure 9:**
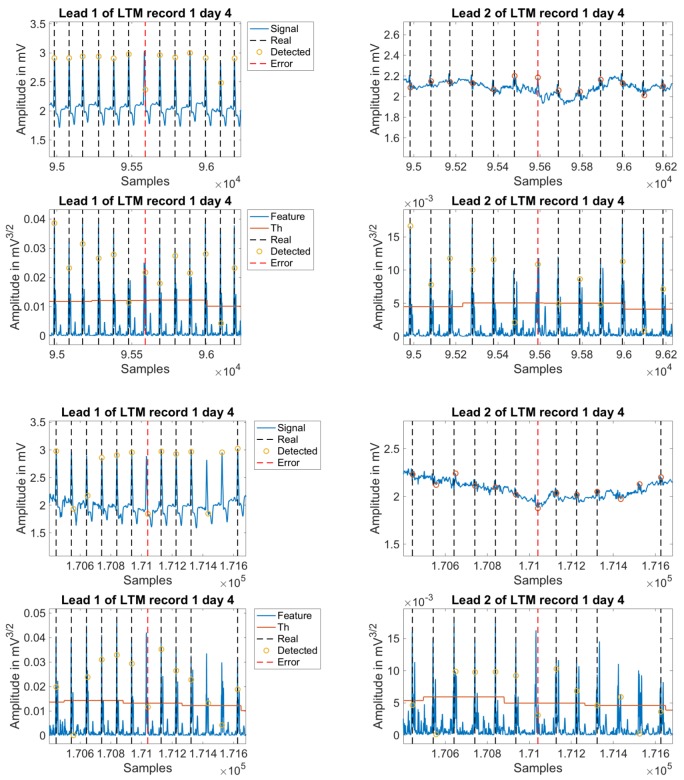
Example of single beat miss-detection in conventional Holter LTM. The panels in the first row show leads 1 and 2 for an example of Holter miss-detection, the second row shows the feature signal for the leads plotted before. The last four panels follow the same scheme for other Holter miss-detection examples.

**Figure 10:**
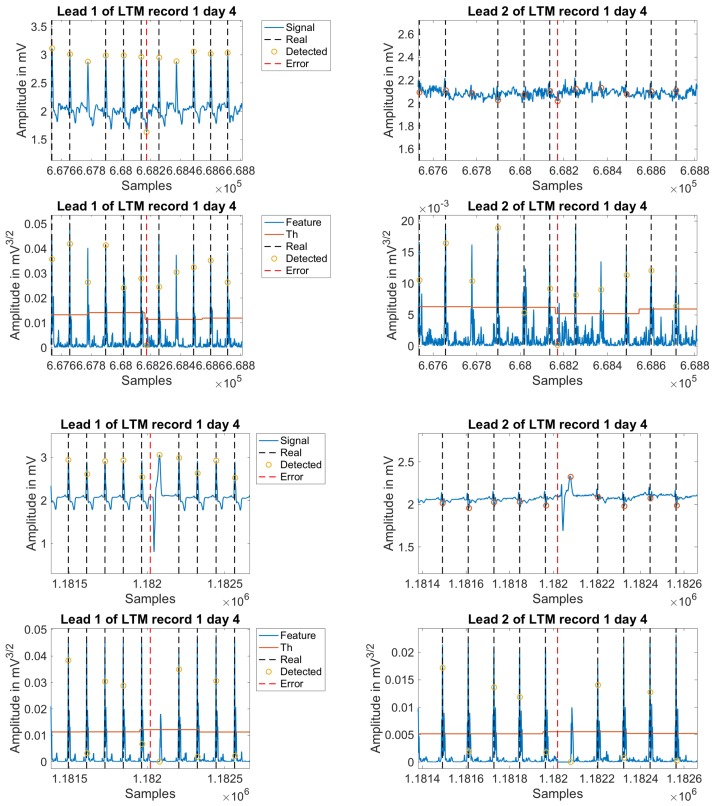
Example of beat miss-detection in proposed algorithm on 7-day Holter recording. The panels in the first row show leads 1 and 2 for an example of algorithm miss-detection, the second row shows the feature signal for the leads plotted before. The last four panels follow the same scheme for other algorithm miss-detection examples.

As previously stated, one of the main purposes of this study was to scrutinize what are the main issues in R-wave detector when applied to LTM. Hence, we manually analyzed the first 500 errors from the worst day in terms of detection errors for each patient, and we classified them as shown in Table 10. On the one hand, those beats that were not detected by the commercial algorithm, but were correctly detected by the proposed algorithm, are referred to as *Holter miss-detection*. Inside this group, we split them into two separate groups, namely, those corresponding to a *Single beat* or those included in a *Burst* of detection errors. On the other hand, those detection errors that were generated by our final algorithm are categorized under the name *Algorithm errors*, regardless they are *FP* or *FN*. As an informative type of identified errors, we included those ones that were not detected either by the commercial software or by our algorithm, and they were called *Shared errors*. The reader can check in Table 10 that about 90% of error detections did not correspond to real errors of the presented algorithm. In addition, within the last 10% of detection errors, up to 5% corresponded to effective errors of the introduced algorithm, and the other 5% corresponded to beats that none of the methods were able to detect. Figure 9 illustrates some examples of error detections in the commercial software, and Figure 10 shows additional examples of error detections by the newly introduced algorithm.

## 5. Discussion and Conclusions

A number of methods and algorithms have been reported in literature for R-wave detection in ECG recordings. A subset including the most relevant R-wave detectors in the literature, as well as new ones based on multilead processing and specially devoted to LTM records, have been proposed, developed, and benchmarked in the present work. LTM algorithms have specific requirements when compared to standard ECG for two reasons. First, the signal processing needs to be extremely efficient to cope with extremely long records (over half a million beats in 7-day recordings), and also it should be simple enough to run on commercial hardware. Second, a much deeper and precise analysis is necessary to extract a clean R-wave, as the signals recorded in LTM incorporate a significantly higher content on noise and other artifacts. A reason for this much worse signal quality is that the recording is not performed in an ambulatory environment. However, even more, the extension over one week or more in LTM, it unfailingly does not facilitate the attention that the patient can make of the system and the electrodes, this becoming an uncomfortable element as the duration extends.

In order to work under these circumstances, an innovative algorithm has been proposed in this paper to handle this new singular environment of LTM, which was compared with the most promising published methods in literature, both over public and private databases. To check its performance in LTM environments, a gold standard database has been labeled by experts and clinicians who manually evaluated two sets of 24-h and 7-day Holter recordings. As a result, not only the designed algorithm improves the detection, but also it incorporates relevant efficient noise removal that can be considered for other Holter and ECG recording types, and not only to LTM.

In order to justify the good behavior of the polling strategy, we can argue that, as a voting strategy itself, and from the statistical point of view, it can be assimilated to median modeling in statistical biological processes. Thus, in our problem, and as in many biological processes where results are better characterized by using a figure of merit that is resilient to single-errors or outliers, the pooling method offers a solid tool to better detect QRS in multilead ECG recordings, as it is ready to overcome singularities, impulsive noise, and other effects that may apply due to the more than frequent uncoupling of electrodes in LTM.

In general, the proposed scheme yields better results than other counterpart detectors in such a way that some of the improvements proposed in this work are not limited to LTM recordings, but they also can be very well applied to conventional Holter. The proposed methods to increase robustness against noise are among the most reliable procedures to hold the performance. Nonetheless, one of the main drawbacks of the current application is the long patient monitoring period itself. Having to deal with the acquisition system during such long time periods becomes uncomfortable and highly tiring for any individual, which thus inevitably reduces the proper care into the system during some of the periods of the seven recording days. This natural disregard causes, therefore and unavoidably, disconnection failures of the electrodes, which are in fact the main cause of errors, and also the reason for which the error rate increases in LTM. Improving the recording systems to be technically more efficient would partially alleviate this situation. However, from an analytical standpoint, a better understanding and characterization of noise is needed to enable a correct delineation of the ECG according to its clinical validity. The elimination of invalid segments from a clinical viewpoint would partially and realistically reduce the error rate in QRS detection. Some works, such as [13], are already facing this problem based on the belief that approaching this issue would provide with room for improvement.

Not all of the categories of proposed ECG analysis algorithms are presented in this work. We only considered those with higher prevalence in the literature, and specially those included on articles dealing with R-wave detection. Other families have not been considered here because they seem not to match with the specific LTM criteria, specially the computational efficiency. As an example, time–frequency analysis methods offer a versatile representation in both the frequency and time domains, by varying the scaled parameters according to global and local signal features, as in the case of detectors based on CWT [31], Discrete Wavelet Transformation [34], STFT [32], HiT [33], or HHT and Empirical Mode Decomposition [35]. The adaptation ability of these methods demand high computational requirements, which currently precludes their use in LTM scenarios. Methods based on advanced statistical and multivariate analysis, like artificial neural networks (ANN), offer high adaptability, but they also demand extended processing time for training. In the field of ECG signal processing, ANN have been proposed as adaptive preprocessing stages [38], R-peak detectors [48], and commonly as classifiers of the detected and segmented QRS complexes [49]. ANN–based detectors deal with ECG signal prediction and examination error; therefore, their extension to LTM scenarios remains to be efficiently formulated.

In the future, we want to scrutinize the improvements of these lately mentioned families of methods and to check what can be expected from them in terms of the preprocessing, feature extraction, and detection logic in LTM scenarios, as a natural continuation of this work. Very recent alternatives are being proposed in the very last generation of works in the literature, which point towards an inflection point in this field. Works in short-term monitoring (duration of some minutes) and new public databases [50] can be found, as the intelligent heart-monitoring public cryptosystem, which involves a patient-worn ECG sensor and a remote monitoring station, using PT and classification trees as heartbeat detection and classification algorithms, respectively. More advanced methods, such as sample entropy, fast Fourier transform, and ANN, were recently selected for the integrated detection algorithms [21], in order to validate an arrhythmia detection method for supra and ventricular ectopic beats and atrial/ventricular fibrillation, when using the ANSI/AAMI EC57:2012 standard. Lossy methods, which traditionally were avoided in this setting, have been successfully revisited [18], and different compression methods based on the compression ratio and percentage root-mean-squared difference were proposed and advantageously benchmarked, including in terms of the accuracy of R-wave detection. Many other works are being developed in the very last years accounting for wavelet multiresolution analysis with promising results [51].

Special attention is being paid to deep learning approaches in ECG monitoring scenarios. In [24], active classification of ECG signals is achieved using a suitable unsupervised feature learning from the raw ECG data, using a softmax regression layer on the top of the resulting hidden representation layer and fine-tuning it with active learning. Their results showed significant accuracy improvements with less expert interaction and faster online retraining compared to existing methods. Other groups [52] showed a deep learning framework to implement a technique for the classification of three selected different cardiac conditions. It was proven to be an efficient automatic arrhythmia detection method while eliminating the burden of training a deep convolutional neural network from scratch. Despite some of these solutions seeming to obviate the need of R-wave detection stages, we should keep in mind that many diagnostic parameters still have to be made on morphological measurements within the delineated ECG, even (specially) in LTM scenarios. Many studies have tested algorithm detection with 24-h Holter recordings, but only some segments of a few seconds or minutes are considered.

Two major issues require attention from researchers when analyzing R-wave detection algorithms. First, the never-ending and growing list of proposed methods in literature almost discourages any attempt to check them all. Second, the difficulty to be able to compare the results as the coding is not described deeply enough in papers, and databases are not always available or shared. This situation is even more complex, as when it comes to LTM cardiac recordings, we are still lacking from a standard and shared framework for comparison. For that reason, in this paper, we first addressed a Longitudinal Optimization, which allowed to benchmark, tune, and improve the best available waveform algorithms in the literature for non-LTM. After that, a modified and multilead R-wave detection algorithm based on PT was proposed with a suitable segmentation and using the complete, and Polling-function combined, content present in all of the available leads. As a consequence, significant improvements were found in terms of accuracy and computational efficiency. In order to get a Transversal Optimization, further analysis and comparisons over public databases were performed to validate and ensure the results’ independence from the tuning databases, sampling frequency, or any other over-fitting effect. Hence, we proved that it is possible to implement an accurate and efficient method, with low computational cost, valid for LTM and suitable for its clinical use. Our 7-day Holter recordings on previous research have been found to be useful in HF patients for the detection of non-sustained atrial or ventricular arrhythmias [26], for the evaluation of heart rate turbulence [53], and for the assessment of autonomic parameters obtained from heart rate variability analysis [25].

## Figures and Tables

**Table 1:** Duration and number of ECGs used from different databases in this study.

Experiment	Database	Type	# ECGs	Duration
Longitudinal	vPREDICT	private	16	24 h
Transversal	MITBIH	public	48	30 m
	NSTDB		15	30 m
	LTSTDB		86	21–24 h
	LTAFDB		84	24–25 h
	LTMITDB		7	14–22 h
	INCART		75	30 m
LTM	PREMARIS	private	5	7 days

**Table 2:** Key QRS detection methods and statistical comparison of their performance over MIT–BIH.

Method	Statistical Measures [%]			Ref.
	SEN	SPE	PPV	
PT	99.75	-	99.53	[9]
HT	99.69	-	99.77	[28]
CWT	99.85	-	99.48	[34]
STFT	99.1	99.6	-	[32]
HiT	99.81	-	99.83	[33]
HHT	98.88	99.04	-	[35]

**Table 3:** Optimal segment lengths, processing time per segment, and detection error for each proposed method in vPREDICT database.

Method	Processing Time (s)	Error (%)	Segment Length (s)
Poll	0.0637	4.05	300
Or	0.0645	4.63	300
And	0.0639	4.05	300
SC	0.0641	3.92	300
ICA	0.1459	26.45	7200
PCA	0.0594	7.27	300
PT	0.0597	12.02	300
RMS	0.0617	4.98	300

**Table 4:** Merit figures using the optimal threshold for each method proposed in the vPREDICT database.

Method	Alpha	Beta	Error (%)
**Poll**	**2.21**	**0.50**	**3.92**
Or	3.93	0.50	4.51
And	2.21	0.50	3.92
SC	2.79	0.50	8.36
ICA	0.50	1.00	47.14
PCA	2.21	0.50	5.93
PT	1.64	0.50	27.75
RMS	0.50	2.00	4.67

**Table 5:** R-wave results on 24-h Holter recordings from vPREDICT and MIT–BIH databases.

vPREDICT	Sen	Spe	PPV	Acc	Error
Mean ± std	99.32 ± 0.96	95.83 ± 3.83	96.99 ± 2.85	97.70 ± 2.08	3.84 ± 3.56
95% CI	[96.49, 99.99]	[88.33, 99.97]	[91.31, 99.98]	[93.45, 99.97]	[0.04, 10.63]
**MIT–BIH**					
Mean ± std	99.01 ± 3.01	99.59 ± 1.53	99.71 ± 1.32	99.19 ± 2.14	1.29 ± 3.40
95% CI	[82.35, 99.96]	[89.87, 100.00]	[90.91, 100.00]	[88.91, 99.98]	[0.04, 12.28]

**Table 6:** Results of the transversal-optimized algorithm using the complete MIT–BIH.

MITBIH					
Record	Sen	Spe	PPV	Acc	Error
100	99.91	100.00	100.00	99.96	0.09
101	99.89	99.87	99.89	99.88	0.21
102	99.95	100.00	100.00	99.98	0.05
103	98.61	99.68	99.90	98.85	1.49
104	99.78	99.79	99.82	99.78	0.40
105	98.64	97.05	98.95	98.16	2.41
106	99.56	99.75	99.85	99.62	0.59
107	99.91	99.95	99.95	99.93	0.14
108	99.49	98.76	99.21	99.05	1.30
109	99.76	100.00	100.00	99.88	0.24
111	99.86	100.00	100.00	99.93	0.14
112	100.00	100.00	100.00	100.00	0.00
113	99.94	100.00	100.00	99.97	0.06
114	99.84	100.00	100.00	99.92	0.16
115	99.69	99.46	99.95	99.67	0.36
116	99.59	99.90	99.92	99.72	0.50
117	99.93	100.00	100.00	99.97	0.07
118	99.96	100.00	100.00	99.98	0.04
119	99.95	99.30	99.95	99.91	0.10
121	100.00	100.00	100.00	100.00	0.00
122	99.96	100.00	100.00	99.98	0.04
123	99.93	100.00	100.00	99.97	0.07
124	99.94	100.00	100.00	99.97	0.06
200	95.42	98.94	99.20	96.88	5.34
201	99.49	100.00	100.00	99.75	0.51
202	99.95	100.00	100.00	99.98	0.05
203	99.26	99.66	99.80	99.41	0.94
205	99.32	100.00	100.00	99.66	0.68
207	99.68	88.89	90.26	94.19	11.08
208	90.66	99.37	99.74	92.96	9.58
209	99.70	99.32	99.93	99.67	0.37
210	99.62	99.92	99.96	99.72	0.42
212	99.78	99.89	99.96	99.81	0.25
213	99.88	100.00	100.00	99.94	0.12
214	99.82	99.94	99.96	99.87	0.22
215	99.97	100.00	100.00	99.99	0.03
217	99.82	100.00	100.00	99.91	0.18
219	99.91	100.00	100.00	99.95	0.09
220	99.95	100.00	100.00	99.98	0.05
221	99.96	100.00	100.00	99.98	0.04
222	99.88	100.00	100.00	99.94	0.12
223	99.58	100.00	100.00	99.79	0.42
228	99.81	99.83	99.95	99.81	0.24
230	99.96	100.00	100.00	99.98	0.04
231	99.94	100.00	100.00	99.97	0.06
232	99.78	98.55	99.16	99.28	1.07
233	99.68	100.00	100.00	99.84	0.32
234	99.93	100.00	100.00	99.96	0.07
**Mean [CI low–CI high]**	**99.48 [90.66–100]**	**99.54 [88.89–100]**	**99.69 [90.26–100]**	**99.46 [92.96–100]**	**0.85 [0–9.58]**

**Table 7:** Results of the transversal-optimized algorithm using the NSTDB.

Record	Sen	Spe	PPV	Acc	Error
118e00	94.56	82.89	89.75	89.61	16.24
118e6	98.29	95.88	97.77	97.29	3.95
118e12	99.74	99.78	99.87	99.72	0.40
118e18	99.96	100.00	100.00	99.98	0.04
118e24	99.96	100.00	100.00	99.98	0.04
118e-6	84.64	75.62	83.14	80.19	32.53
119e00	96.02	81.86	88.70	89.92	16.21
119e06	99.45	95.38	97.34	97.82	3.27
119e12	99.95	98.88	99.90	99.86	0.15
119e18	99.95	99.30	99.95	99.91	0.10
119e24	99.95	99.30	99.95	99.91	0.10
119e-6	89.28	71.09	80.20	80.76	32.76

**Table 8:** Results of the transversal-optimized algorithm using non-LTM databases.

Database	Sen	Spe	PPV	Acc	Error
vPREDICT	99.55 [98.06–99.99]	95.64 [87.53–99.97]	96.87 [90.63–99.98]	97.77 [93.58–99.98]	3.75 [0.03–10.56]
LTAFDB	98.89 [91.08–99.99]	96.38 [80.96–99.95]	97.56 [88.71–99.96]	97.69 [91.01–99.92]	3.66 [0.07–12.92]
LTSTDB	99.88 [99.19–100]	99.80 [97.49–100]	99.87 [98.74–100]	99.84 [98.54–100]	0.25 [0.00–1.87]
LTMITDB	99.68 [98.86–99.99]	98.52 [96.57–99.97]	99.02 [97.62–99.98]	99.16 [98.25–99.98]	1.32 [0.03–2.86]
INCART	99.36 [100–95.33]	99.63 [96.64–100]	99.78 [98.00–100]	99.46 [96.92–100]	0.86 [0–4.89]

**Table 9:** Results of the optimized algorithm on 7-day Holter database PREMARIS.

Patient	SEN	SPE	PPV	ACC	ERR
1	99.32	91.00	93.46	95.46	7.70
2	99.44	88.32	91.04	94.09	11.05
3	99.87	95.32	96.61	97.77	3.72
4	99.90	96.30	97.22	98.26	2.97
5	99.98	98.67	99.09	99.43	0.94
**Mean [CI low–CI high]**	99.70 [99.98–53.43]	98.67 [93.92–60.52]	99.09 [95.48–57.61]	99.43 [97–55.94]	86.89 [5.28–0.94]

**Table 10:** Results of analyzing the first 500 errors in the worst day of the LTM recordings in PREMARIS database.

		Holter Miss-Detections		Algorithm Errors		Shared Errors
Patient	Analyzed Segments (h)	Burst	Single Beat	FP	FN	
1	4.23	217	233	25	15	10
2	3.5	449	17	10	10	14
3	2.54	59	335	19	18	69
4	5.32	118	342	2	13	25
5	7.52	37	462	0	1	0
